# (6-Hydroxy-2-{[2-(*N*-methyl­carbamo­thiol­yl)hydrazin-1-yl­idene-κ^2^ *N* ^1^,*S*]meth­yl}­phenolato-κ*O* ^1^)(triphenyl­phosphane-κ*P*)nickel(II) chloride

**DOI:** 10.1107/S1600536810039292

**Published:** 2010-10-09

**Authors:** Hana Bashir Shawish, M. Jamil Maah, Seik Weng Ng

**Affiliations:** a Department of Chemistry, University of Malaya, 50603 Kuala Lumpur, Malaysia

## Abstract

The deprotonated Schiff base ligand in the title salt, [Ni(C_9_H_10_N_3_O_2_S)(C_18_H_15_P)]Cl, functions as an *N*,*O*,*S*-chelating anion to the phosphine-coordinated nickel(II) atom, which exists in a distorted square-planar geometry. The hy­droxy group forms an intra­molecular O—H⋯O hydrogen bond. The two amino groups of the cation are hydrogen-bond donors to the chloride anion; the hydrogen bonds generate a chain structure running along the *b* axis.

## Related literature

The only report of this Schiff base is that of a study of its organotin derivatives; see: Swesi *et al.* (2007 ▶). For a related nickel Schiff-base adduct of triphenyl­phosphine, see: Shawish *et al.* (2010 ▶).
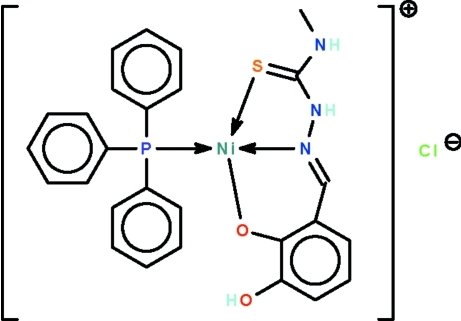


## Experimental

### 

#### Crystal data

[Ni(C_9_H_10_N_3_O_2_S)(C_18_H_15_P)]Cl
*M* *_r_* = 580.69
Monoclinic, 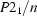

*a* = 15.7781 (15) Å
*b* = 10.6306 (10) Å
*c* = 17.0020 (15) Å
β = 113.961 (1)°
*V* = 2606.0 (4) Å^3^
*Z* = 4
Mo *K*α radiation
μ = 1.02 mm^−1^
*T* = 100 K
0.25 × 0.15 × 0.05 mm

#### Data collection

Bruker SMART APEX diffractometer
Absorption correction: multi-scan (*SADABS*; Sheldrick, 1996 ▶) *T*_min_ = 0.785, *T*_max_ = 0.951
23696 measured reflections
5971 independent reflections
4428 reflections with *I* > 2σ(*I*)
*R*_int_ = 0.096

#### Refinement

*R*[*F* ^2^ > 2σ(*F* ^2^)] = 0.061
*wR*(*F* ^2^) = 0.177
*S* = 1.05
5971 reflections
338 parameters
3 restraints
H atoms treated by a mixture of independent and constrained refinement
Δρ_max_ = 1.60 e Å^−3^
Δρ_min_ = −1.06 e Å^−3^

### 

Data collection: *APEX2* (Bruker, 2009 ▶); cell refinement: *SAINT* (Bruker, 2009 ▶); data reduction: *SAINT*; program(s) used to solve structure: *SHELXS97* (Sheldrick, 2008 ▶); program(s) used to refine structure: *SHELXL97* (Sheldrick, 2008 ▶); molecular graphics: *X-SEED* (Barbour, 2001 ▶); software used to prepare material for publication: *publCIF* (Westrip, 2010 ▶).

## Supplementary Material

Crystal structure: contains datablocks global, I. DOI: 10.1107/S1600536810039292/xu5041sup1.cif

Structure factors: contains datablocks I. DOI: 10.1107/S1600536810039292/xu5041Isup2.hkl

Additional supplementary materials:  crystallographic information; 3D view; checkCIF report

## Figures and Tables

**Table 1:** Selected bond lengths (Å)

Ni1—N1	1.895 (3)
Ni1—O1	1.849 (2)
Ni1—P1	2.216 (1)
Ni1—S1	2.150 (1)

**Table 2:** Hydrogen-bond geometry (Å, °)

*D*—H⋯*A*	*D*—H	H⋯*A*	*D*⋯*A*	*D*—H⋯*A*
O2—H2*O*⋯O1	0.84 (5)	2.10 (4)	2.640 (4)	122 (4)
N2—H2*N*⋯Cl1	0.86 (4)	2.19 (4)	3.046 (3)	172 (5)
N3—H3*N*⋯Cl1^i^	0.86 (4)	2.28 (4)	3.111 (3)	164 (5)

Symmetry code: (i) 
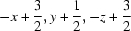
.

**Fig. 1.**
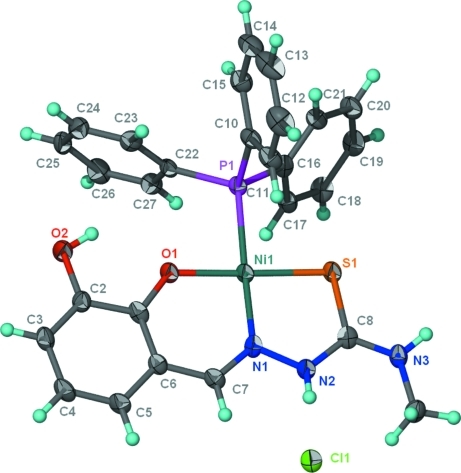
Thermal ellipsoid plot (Barbour, 2001) of Ni(C9H10N3O2S)(C18H15P).Cl at the 70% probability level; hydrogen atoms are drawn as spheres of arbitrary radius.

**Fig. 2.**
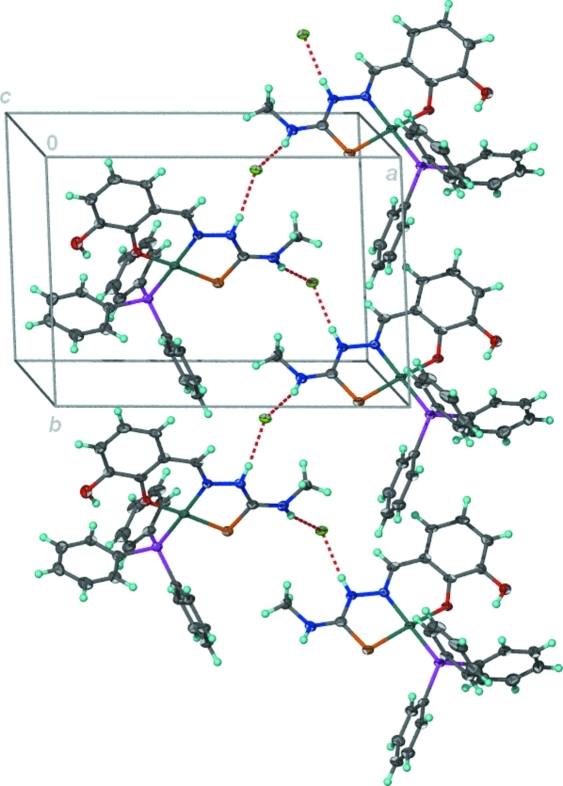
Hydrogen-bonded chain motif.

## supplementary crystallographic information

### Experimental

2,3-Dihydroxybenzaldehyde 4-methylthiosemicarbazone hemihydrate (Swesi *et al.*, 2007) (0.22 g, 1 mmol), triphenylphosphine (0.26, 1 mmol) and nickel chloride (0.13 g, 1 mmol) were heated in a methanol/ethanol (50 ml) for an hour. The brown solution was then set aside for the growth of crystals.

### Refinement

Carbon-bound H-atoms were placed in calculated positions (C—H 0.95–0.98 Å) and were included in the refinement in the riding model approximation, with *U*(H) set to 1.2–1.5*U*(C).

The amino and hydroxy H-atoms were located in a difference Fourier map, and were refined with distance restraints of N–H 0.86±0.01 and O–H 0.84±0.01 Å; their temperature factors were freely refined.

### Crystal data

**Table:**

[Ni(C_9_H_10_N_3_O_2_S)(C_18_H_15_P)]Cl	*F*(000) = 1200
*M**_r_* = 580.69	*D*_x_ = 1.480 Mg m^−^^3^
Monoclinic, *P*2_1_/*n*	Mo *K*α radiation, λ = 0.71073 Å
Hall symbol: -P 2yn	Cell parameters from 3978 reflections
*a* = 15.7781 (15) Å	θ = 2.3–27.6°
*b* = 10.6306 (10) Å	µ = 1.02 mm^−^^1^
*c* = 17.0020 (15) Å	*T* = 100 K
β = 113.961 (1)°	Triangular block, brown
*V* = 2606.0 (4) Å^3^	0.25 × 0.15 × 0.05 mm
*Z* = 4	

### Data collection

**Table:**

Bruker SMART APEX diffractometer	5971 independent reflections
Radiation source: fine-focus sealed tube	4428 reflections with *I* > 2σ(*I*)
graphite	*R*_int_ = 0.096
ω scans	θ_max_ = 27.5°, θ_min_ = 2.3°
Absorption correction: multi-scan (*SADABS*; Sheldrick, 1996)	*h* = −20→20
*T*_min_ = 0.785, *T*_max_ = 0.951	*k* = −13→13
23696 measured reflections	*l* = −22→21

### Refinement

**Table:**

Refinement on *F*^2^	Primary atom site location: structure-invariant direct methods
Least-squares matrix: full	Secondary atom site location: difference Fourier map
*R*[*F*^2^ > 2σ(*F*^2^)] = 0.061	Hydrogen site location: inferred from neighbouring sites
*wR*(*F*^2^) = 0.177	H atoms treated by a mixture of independent and constrained refinement
*S* = 1.05	*w* = 1/[σ^2^(*F*_o_^2^) + (0.1036*P*)^2^ + 0.1986*P*] where *P* = (*F*_o_^2^ + 2*F*_c_^2^)/3
5971 reflections	(Δ/σ)_max_ = 0.001
338 parameters	Δρ_max_ = 1.60 e Å^−^^3^
3 restraints	Δρ_min_ = −1.06 e Å^−^^3^

### Fractional atomic coordinates and isotropic or equivalent isotropic displacement parameters (Å^2^)

**Table:**

	*x*	*y*	*z*	*U*_iso_*/*U*_eq_	
Ni1	0.41946 (3)	0.54484 (4)	0.60995 (3)	0.01585 (16)	
Cl1	0.66145 (6)	0.16147 (8)	0.61950 (6)	0.0205 (2)	
S1	0.55028 (6)	0.62917 (8)	0.68921 (6)	0.0200 (2)	
P1	0.34464 (6)	0.68668 (9)	0.65317 (6)	0.0162 (2)	
O1	0.30452 (17)	0.4812 (2)	0.53950 (17)	0.0194 (6)	
O2	0.12753 (18)	0.4272 (3)	0.45169 (19)	0.0268 (6)	
H2O	0.159 (3)	0.481 (4)	0.488 (3)	0.047 (16)*	
N1	0.4861 (2)	0.4179 (3)	0.58096 (19)	0.0170 (6)	
N2	0.5828 (2)	0.4237 (3)	0.6177 (2)	0.0183 (6)	
H2N	0.609 (3)	0.353 (3)	0.616 (4)	0.060 (18)*	
N3	0.7127 (2)	0.5302 (3)	0.7092 (2)	0.0194 (7)	
H3N	0.739 (3)	0.577 (4)	0.753 (2)	0.042 (15)*	
C1	0.2882 (2)	0.3775 (3)	0.4925 (2)	0.0185 (7)	
C2	0.1937 (2)	0.3465 (3)	0.4458 (2)	0.0194 (8)	
C3	0.1667 (3)	0.2402 (4)	0.3963 (3)	0.0226 (8)	
H3	0.1027	0.2211	0.3665	0.027*	
C4	0.2342 (3)	0.1597 (3)	0.3899 (3)	0.0230 (8)	
H4	0.2156	0.0854	0.3561	0.028*	
C5	0.3271 (3)	0.1876 (3)	0.4321 (2)	0.0208 (8)	
H5	0.3723	0.1340	0.4262	0.025*	
C6	0.3553 (2)	0.2978 (3)	0.4851 (2)	0.0178 (7)	
C7	0.4516 (3)	0.3234 (3)	0.5293 (2)	0.0187 (7)	
H7	0.4938	0.2674	0.5205	0.022*	
C8	0.6223 (2)	0.5188 (3)	0.6721 (2)	0.0180 (7)	
C9	0.7785 (3)	0.4427 (4)	0.6972 (3)	0.0231 (8)	
H9A	0.8413	0.4775	0.7242	0.035*	
H9B	0.7618	0.4306	0.6356	0.035*	
H9C	0.7763	0.3617	0.7238	0.035*	
C10	0.3352 (3)	0.6414 (3)	0.7521 (2)	0.0197 (8)	
C11	0.3826 (3)	0.5350 (3)	0.7982 (3)	0.0236 (8)	
H11	0.4201	0.4865	0.7778	0.028*	
C12	0.3746 (3)	0.5011 (4)	0.8736 (3)	0.0325 (10)	
H12	0.4075	0.4300	0.9052	0.039*	
C13	0.3190 (3)	0.5697 (4)	0.9031 (3)	0.0342 (10)	
H13	0.3130	0.5445	0.9542	0.041*	
C14	0.2719 (3)	0.6755 (4)	0.8584 (3)	0.0299 (9)	
H14	0.2345	0.7233	0.8793	0.036*	
C15	0.2798 (3)	0.7105 (4)	0.7838 (3)	0.0242 (8)	
H15	0.2473	0.7825	0.7532	0.029*	
C16	0.3967 (2)	0.8436 (3)	0.6713 (2)	0.0187 (7)	
C17	0.4240 (2)	0.8905 (4)	0.6092 (2)	0.0208 (8)	
H17	0.4146	0.8416	0.5596	0.025*	
C18	0.4653 (3)	1.0087 (4)	0.6187 (3)	0.0229 (8)	
H18	0.4827	1.0410	0.5753	0.028*	
C19	0.4808 (3)	1.0785 (4)	0.6917 (3)	0.0253 (8)	
H19	0.5093	1.1588	0.6988	0.030*	
C20	0.4549 (3)	1.0321 (4)	0.7544 (3)	0.0249 (9)	
H20	0.4655	1.0810	0.8043	0.030*	
C21	0.4132 (2)	0.9143 (4)	0.7454 (2)	0.0205 (8)	
H21	0.3963	0.8824	0.7892	0.025*	
C22	0.2247 (2)	0.7088 (3)	0.5762 (2)	0.0186 (7)	
C23	0.1512 (3)	0.6499 (4)	0.5882 (3)	0.0219 (8)	
H23	0.1636	0.5983	0.6373	0.026*	
C24	0.0605 (3)	0.6665 (4)	0.5290 (3)	0.0255 (9)	
H24	0.0110	0.6277	0.5380	0.031*	
C25	0.0422 (3)	0.7399 (4)	0.4563 (3)	0.0250 (9)	
H25	−0.0199	0.7524	0.4159	0.030*	
C26	0.1149 (3)	0.7950 (4)	0.4429 (3)	0.0279 (9)	
H26	0.1023	0.8435	0.3924	0.033*	
C27	0.2057 (3)	0.7801 (4)	0.5021 (2)	0.0218 (8)	
H27	0.2549	0.8185	0.4922	0.026*	

### Atomic displacement parameters (Å^2^)

**Table:**

	*U*^11^	*U*^22^	*U*^33^	*U*^12^	*U*^13^	*U*^23^
Ni1	0.0167 (3)	0.0092 (3)	0.0194 (3)	−0.00037 (16)	0.0050 (2)	−0.00177 (17)
Cl1	0.0221 (4)	0.0127 (4)	0.0235 (5)	0.0019 (3)	0.0060 (4)	0.0012 (3)
S1	0.0185 (4)	0.0130 (4)	0.0248 (5)	−0.0006 (3)	0.0051 (4)	−0.0051 (4)
P1	0.0178 (4)	0.0110 (4)	0.0183 (5)	−0.0007 (3)	0.0058 (4)	−0.0012 (4)
O1	0.0177 (12)	0.0122 (13)	0.0250 (14)	−0.0008 (10)	0.0054 (11)	−0.0056 (10)
O2	0.0198 (13)	0.0210 (15)	0.0362 (17)	−0.0019 (11)	0.0080 (12)	−0.0132 (13)
N1	0.0157 (14)	0.0116 (14)	0.0200 (16)	−0.0006 (11)	0.0034 (12)	0.0024 (12)
N2	0.0172 (15)	0.0120 (15)	0.0227 (17)	0.0010 (12)	0.0049 (13)	−0.0024 (13)
N3	0.0180 (15)	0.0152 (16)	0.0213 (17)	−0.0026 (12)	0.0041 (13)	0.0000 (13)
C1	0.0243 (18)	0.0105 (17)	0.0180 (18)	−0.0020 (14)	0.0057 (15)	0.0001 (14)
C2	0.0174 (17)	0.0165 (18)	0.0221 (19)	−0.0011 (14)	0.0058 (15)	−0.0016 (15)
C3	0.0205 (17)	0.0198 (19)	0.023 (2)	−0.0044 (15)	0.0041 (15)	−0.0044 (16)
C4	0.029 (2)	0.0123 (18)	0.024 (2)	−0.0029 (15)	0.0067 (16)	−0.0063 (15)
C5	0.0253 (19)	0.0107 (17)	0.024 (2)	0.0030 (14)	0.0070 (16)	−0.0024 (15)
C6	0.0205 (17)	0.0145 (18)	0.0170 (18)	−0.0014 (13)	0.0063 (15)	−0.0001 (14)
C7	0.0236 (18)	0.0126 (17)	0.0176 (19)	0.0026 (14)	0.0058 (15)	0.0006 (14)
C8	0.0203 (17)	0.0131 (17)	0.0186 (18)	0.0006 (14)	0.0057 (15)	0.0047 (14)
C9	0.0187 (18)	0.0173 (19)	0.030 (2)	0.0020 (14)	0.0070 (16)	−0.0015 (16)
C10	0.0255 (18)	0.0146 (18)	0.0196 (19)	−0.0047 (14)	0.0098 (16)	−0.0039 (15)
C11	0.031 (2)	0.0152 (19)	0.023 (2)	−0.0004 (15)	0.0088 (17)	−0.0022 (15)
C12	0.046 (3)	0.019 (2)	0.031 (2)	−0.0069 (19)	0.014 (2)	0.0020 (18)
C13	0.052 (3)	0.028 (2)	0.027 (2)	−0.016 (2)	0.021 (2)	−0.0039 (19)
C14	0.037 (2)	0.030 (2)	0.027 (2)	−0.0101 (18)	0.0175 (19)	−0.0093 (18)
C15	0.0259 (19)	0.019 (2)	0.027 (2)	−0.0037 (15)	0.0105 (17)	−0.0042 (16)
C16	0.0175 (17)	0.0104 (17)	0.024 (2)	0.0028 (13)	0.0037 (15)	0.0021 (14)
C17	0.0222 (18)	0.0151 (19)	0.023 (2)	−0.0037 (14)	0.0070 (16)	−0.0021 (15)
C18	0.0255 (19)	0.0163 (19)	0.026 (2)	−0.0020 (15)	0.0091 (16)	0.0058 (16)
C19	0.0246 (19)	0.0144 (18)	0.033 (2)	−0.0032 (15)	0.0083 (17)	−0.0002 (17)
C20	0.027 (2)	0.018 (2)	0.027 (2)	−0.0009 (15)	0.0078 (17)	−0.0056 (16)
C21	0.0233 (18)	0.0164 (18)	0.022 (2)	−0.0010 (14)	0.0088 (15)	−0.0021 (15)
C22	0.0183 (17)	0.0152 (18)	0.0187 (19)	0.0013 (14)	0.0039 (14)	−0.0060 (14)
C23	0.0232 (18)	0.0143 (18)	0.028 (2)	0.0011 (14)	0.0107 (16)	−0.0017 (16)
C24	0.0212 (19)	0.022 (2)	0.033 (2)	−0.0039 (15)	0.0106 (17)	−0.0085 (17)
C25	0.0175 (17)	0.025 (2)	0.025 (2)	0.0038 (15)	0.0007 (15)	−0.0066 (17)
C26	0.030 (2)	0.022 (2)	0.024 (2)	0.0044 (16)	0.0042 (17)	0.0000 (17)
C27	0.0228 (18)	0.021 (2)	0.020 (2)	0.0019 (15)	0.0077 (15)	0.0003 (15)

### Geometric parameters (Å, °)

**Table:**

Ni1—N1	1.895 (3)	C10—C15	1.406 (5)
Ni1—O1	1.849 (2)	C11—C12	1.385 (6)
Ni1—P1	2.216 (1)	C11—H11	0.9500
Ni1—S1	2.150 (1)	C12—C13	1.382 (7)
S1—C8	1.738 (4)	C12—H12	0.9500
P1—C10	1.813 (4)	C13—C14	1.391 (7)
P1—C22	1.826 (4)	C13—H13	0.9500
P1—C16	1.830 (4)	C14—C15	1.376 (6)
O1—C1	1.324 (4)	C14—H14	0.9500
O2—C2	1.386 (4)	C15—H15	0.9500
O2—H2O	0.84 (5)	C16—C17	1.385 (5)
N1—C7	1.299 (5)	C16—C21	1.397 (5)
N1—N2	1.396 (4)	C17—C18	1.394 (5)
N2—C8	1.341 (5)	C17—H17	0.9500
N2—H2N	0.86 (4)	C18—C19	1.380 (6)
N3—C8	1.311 (5)	C18—H18	0.9500
N3—C9	1.468 (5)	C19—C20	1.378 (6)
N3—H3N	0.86 (4)	C19—H19	0.9500
C1—C6	1.401 (5)	C20—C21	1.393 (5)
C1—C2	1.415 (5)	C20—H20	0.9500
C2—C3	1.369 (5)	C21—H21	0.9500
C3—C4	1.405 (5)	C22—C27	1.395 (5)
C3—H3	0.9500	C22—C23	1.405 (5)
C4—C5	1.377 (5)	C23—C24	1.386 (5)
C4—H4	0.9500	C23—H23	0.9500
C5—C6	1.434 (5)	C24—C25	1.389 (6)
C5—H5	0.9500	C24—H24	0.9500
C6—C7	1.422 (5)	C25—C26	1.387 (6)
C7—H7	0.9500	C25—H25	0.9500
C9—H9A	0.9800	C26—C27	1.385 (5)
C9—H9B	0.9800	C26—H26	0.9500
C9—H9C	0.9800	C27—H27	0.9500
C10—C11	1.406 (5)		
			
O1—Ni1—N1	94.19 (12)	C11—C10—C15	118.6 (4)
O1—Ni1—S1	176.80 (9)	C11—C10—P1	120.4 (3)
N1—Ni1—S1	88.09 (9)	C15—C10—P1	121.0 (3)
O1—Ni1—P1	87.10 (8)	C12—C11—C10	119.9 (4)
N1—Ni1—P1	175.83 (10)	C12—C11—H11	120.1
S1—Ni1—P1	90.78 (4)	C10—C11—H11	120.1
C8—S1—Ni1	98.04 (13)	C13—C12—C11	120.5 (4)
C10—P1—C22	104.30 (17)	C13—C12—H12	119.7
C10—P1—C16	106.66 (18)	C11—C12—H12	119.7
C22—P1—C16	105.45 (17)	C12—C13—C14	120.3 (4)
C10—P1—Ni1	112.48 (13)	C12—C13—H13	119.8
C22—P1—Ni1	112.74 (12)	C14—C13—H13	119.8
C16—P1—Ni1	114.42 (12)	C15—C14—C13	119.6 (4)
C1—O1—Ni1	126.5 (2)	C15—C14—H14	120.2
C2—O2—H2O	104 (4)	C13—C14—H14	120.2
C7—N1—N2	114.7 (3)	C14—C15—C10	121.0 (4)
C7—N1—Ni1	127.0 (3)	C14—C15—H15	119.5
N2—N1—Ni1	118.3 (2)	C10—C15—H15	119.5
C8—N2—N1	117.3 (3)	C17—C16—C21	119.4 (3)
C8—N2—H2N	125 (4)	C17—C16—P1	117.2 (3)
N1—N2—H2N	114 (4)	C21—C16—P1	123.3 (3)
C8—N3—C9	124.6 (3)	C16—C17—C18	120.8 (4)
C8—N3—H3N	121 (3)	C16—C17—H17	119.6
C9—N3—H3N	112 (3)	C18—C17—H17	119.6
O1—C1—C6	126.1 (3)	C19—C18—C17	119.4 (4)
O1—C1—C2	115.8 (3)	C19—C18—H18	120.3
C6—C1—C2	118.1 (3)	C17—C18—H18	120.3
C3—C2—O2	120.0 (3)	C20—C19—C18	120.3 (4)
C3—C2—C1	122.1 (3)	C20—C19—H19	119.9
O2—C2—C1	118.0 (3)	C18—C19—H19	119.9
C2—C3—C4	119.6 (3)	C19—C20—C21	120.7 (4)
C2—C3—H3	120.2	C19—C20—H20	119.6
C4—C3—H3	120.2	C21—C20—H20	119.6
C5—C4—C3	120.5 (3)	C20—C21—C16	119.3 (4)
C5—C4—H4	119.7	C20—C21—H21	120.3
C3—C4—H4	119.7	C16—C21—H21	120.3
C4—C5—C6	119.8 (3)	C27—C22—C23	119.1 (3)
C4—C5—H5	120.1	C27—C22—P1	119.8 (3)
C6—C5—H5	120.1	C23—C22—P1	121.0 (3)
C1—C6—C7	121.4 (3)	C24—C23—C22	120.5 (4)
C1—C6—C5	119.8 (3)	C24—C23—H23	119.8
C7—C6—C5	118.8 (3)	C22—C23—H23	119.8
N1—C7—C6	124.8 (3)	C23—C24—C25	119.9 (4)
N1—C7—H7	117.6	C23—C24—H24	120.0
C6—C7—H7	117.6	C25—C24—H24	120.0
N3—C8—N2	120.7 (3)	C26—C25—C24	119.7 (3)
N3—C8—S1	121.0 (3)	C26—C25—H25	120.1
N2—C8—S1	118.2 (3)	C24—C25—H25	120.1
N3—C9—H9A	109.5	C27—C26—C25	120.8 (4)
N3—C9—H9B	109.5	C27—C26—H26	119.6
H9A—C9—H9B	109.5	C25—C26—H26	119.6
N3—C9—H9C	109.5	C26—C27—C22	119.9 (4)
H9A—C9—H9C	109.5	C26—C27—H27	120.0
H9B—C9—H9C	109.5	C22—C27—H27	120.0
			
N1—Ni1—S1—C8	−1.59 (15)	C22—P1—C10—C11	130.3 (3)
P1—Ni1—S1—C8	174.39 (13)	C16—P1—C10—C11	−118.5 (3)
O1—Ni1—P1—C10	95.87 (15)	Ni1—P1—C10—C11	7.8 (3)
S1—Ni1—P1—C10	−86.52 (13)	C22—P1—C10—C15	−49.1 (3)
O1—Ni1—P1—C22	−21.73 (16)	C16—P1—C10—C15	62.2 (3)
S1—Ni1—P1—C22	155.87 (14)	Ni1—P1—C10—C15	−171.6 (3)
O1—Ni1—P1—C16	−142.22 (16)	C15—C10—C11—C12	−0.5 (6)
S1—Ni1—P1—C16	35.38 (14)	P1—C10—C11—C12	−179.8 (3)
N1—Ni1—O1—C1	2.5 (3)	C10—C11—C12—C13	1.1 (6)
P1—Ni1—O1—C1	−173.5 (3)	C11—C12—C13—C14	−1.4 (7)
O1—Ni1—N1—C7	−1.3 (3)	C12—C13—C14—C15	1.0 (6)
S1—Ni1—N1—C7	−179.0 (3)	C13—C14—C15—C10	−0.3 (6)
O1—Ni1—N1—N2	178.6 (3)	C11—C10—C15—C14	0.1 (6)
S1—Ni1—N1—N2	0.8 (2)	P1—C10—C15—C14	179.4 (3)
C7—N1—N2—C8	−179.4 (3)	C10—P1—C16—C17	170.3 (3)
Ni1—N1—N2—C8	0.7 (4)	C22—P1—C16—C17	−79.2 (3)
Ni1—O1—C1—C6	−2.1 (5)	Ni1—P1—C16—C17	45.3 (3)
Ni1—O1—C1—C2	178.1 (3)	C10—P1—C16—C21	−6.8 (4)
O1—C1—C2—C3	−178.3 (4)	C22—P1—C16—C21	103.7 (3)
C6—C1—C2—C3	2.0 (6)	Ni1—P1—C16—C21	−131.9 (3)
O1—C1—C2—O2	1.3 (5)	C21—C16—C17—C18	−1.9 (5)
C6—C1—C2—O2	−178.5 (3)	P1—C16—C17—C18	−179.2 (3)
O2—C2—C3—C4	179.2 (4)	C16—C17—C18—C19	1.4 (6)
C1—C2—C3—C4	−1.2 (6)	C17—C18—C19—C20	−0.5 (6)
C2—C3—C4—C5	−0.7 (6)	C18—C19—C20—C21	0.3 (6)
C3—C4—C5—C6	1.8 (6)	C19—C20—C21—C16	−0.8 (6)
O1—C1—C6—C7	−0.4 (6)	C17—C16—C21—C20	1.6 (5)
C2—C1—C6—C7	179.3 (3)	P1—C16—C21—C20	178.7 (3)
O1—C1—C6—C5	179.4 (3)	C10—P1—C22—C27	160.2 (3)
C2—C1—C6—C5	−0.9 (5)	C16—P1—C22—C27	48.1 (3)
C4—C5—C6—C1	−0.9 (6)	Ni1—P1—C22—C27	−77.4 (3)
C4—C5—C6—C7	178.8 (4)	C10—P1—C22—C23	−23.0 (3)
N2—N1—C7—C6	179.6 (3)	C16—P1—C22—C23	−135.1 (3)
Ni1—N1—C7—C6	−0.6 (5)	Ni1—P1—C22—C23	99.4 (3)
C1—C6—C7—N1	1.8 (6)	C27—C22—C23—C24	−2.6 (5)
C5—C6—C7—N1	−178.0 (3)	P1—C22—C23—C24	−179.4 (3)
C9—N3—C8—N2	−1.2 (6)	C22—C23—C24—C25	1.2 (6)
C9—N3—C8—S1	−178.9 (3)	C23—C24—C25—C26	0.8 (6)
N1—N2—C8—N3	179.9 (3)	C24—C25—C26—C27	−1.5 (6)
N1—N2—C8—S1	−2.3 (4)	C25—C26—C27—C22	0.1 (6)
Ni1—S1—C8—N3	−179.7 (3)	C23—C22—C27—C26	1.9 (6)
Ni1—S1—C8—N2	2.5 (3)	P1—C22—C27—C26	178.7 (3)

### Hydrogen-bond geometry (Å, °)

**Table:**

*D*—H···*A*	*D*—H	H···*A*	*D*···*A*	*D*—H···*A*
O2—H2O···O1	0.84 (5)	2.10 (4)	2.640 (4)	122 (4)
N2—H2N···Cl1	0.86 (4)	2.19 (4)	3.046 (3)	172 (5)
N3—H3N···Cl1^i^	0.86 (4)	2.28 (4)	3.111 (3)	164 (5)

Symmetry codes: (i) −*x*+3/2, *y*+1/2, −*z*+3/2.
